# Integrating surface electromyography into physical therapy training with the support of STEM education

**DOI:** 10.3389/fneur.2024.1501619

**Published:** 2024-11-21

**Authors:** Xing-kai Liu, Yu Qu, Kimiko Tajiri, Ping Zhou, Ming Huo

**Affiliations:** ^1^Department of Rehabilitation Medicine, Xijing Hospital, Fourth Military Medical University, Xi'an, Shaanxi, China; ^2^Qilu Hospital of Shandong University, Jinan, China; ^3^School of Rehabilitation Sciences and Engineering, University of Health and Rehabilitation Sciences, Qingdao, China; ^4^Department of Nursing, School of Health Sciences, Bukkyo University, Kyoto, Japan; ^5^Qingdao Hospital, University of Health and Rehabilitation Sciences (Qingdao Municipal Hospital), Qingdao, China

**Keywords:** surface electromyography (SEMG), physical therapy education, STEM education framework, teaching strategies, clinical competencies

## 1 Introduction

In the context of 21st-century innovations in education, the STEM (Science, Technology, Engineering, and Mathematics) educational framework facilitates the integration of interdisciplinary knowledge and fosters innovative thinking, playing a pivotal role particularly in the field of health sciences (1). Physical therapy, as a vital branch of health sciences, starts to proactively embrace STEM integration in education in several countries, and is dedicated to training professionals with a broad spectrum of skills. For instance, undergraduate physical therapy programs in Chile have significantly enhanced students' comprehension of human movement by integrating sEMG technology and active learning strategies (2). Additionally, a free 3-day event with sEMG theoretical classes and computer programming in Matlab in Brazil has markedly improved participants' understanding of sEMG through an integrated approach combining theoretical and practical course content (3). In particular, the integration, teaching, and application of sEMG technology within the STEM education framework offers new perspectives and innovative methodologies for physical therapy education. Beyond sEMG, which serves as one of fundamental components of evidence-based practice, other technologies, such as virtual reality (VR), augmented reality (AR), 3D printing, and telehealth etc., are increasingly being utilized to enhance STEM literacy in physical therapy education. VR and AR technologies, for instance, offer students a safe environment to practice their assessment and treatment skills through simulated clinical scenarios (4). The application of 3D printing in education, such as creating anatomical models, aids students in better understanding human anatomy, exemplifying the practical application of engineering and mathematical principles in STEM education (2). Furthermore, telehealth technologies require physical therapists to interpret and analyze data remotely, underscoring the significance of technological proficiency in modern rehabilitation practices (5).

As a non-invasive neurophysiological diagnostic tool, sEMG technology serves as a potent instrument for real-time monitoring and quantitative analysis of muscle function, which is a critical aspect of advancing the clinical skills of physical therapists (6). However, the optimal use of sEMG technology requires the development of novel approaches to curriculum content and teaching methods in physical therapy education.

This article aims to examine the application of sEMG technology in physical therapy education within the STEM education framework, to scrutinize the pedagogical challenges in teaching implementation, and to delineate potential strategies to address these challenges. The objectives of this communication are threefold: to promote educational innovation, to enhance curriculum design, and to provide theoretical and practical guidance for the cultivation of physical therapy professionals endowed with STEM literacy.

## 2 Integrating sEMG technology with STEM Disciplines IN physical therapy education: an overview

Surface electromyography (sEMG) technology has been broadly applied in the field of physical therapy. By placing electrodes on the skin's surface, sEMG can capture the electrical signals generated by muscle activity. These signals, in turn, reflect the activity of muscle fibers, enabling the analysis of muscle function and control strategies (7). Merletti (8) highlighted the pivotal role of sEMG technology as a fundamental component of evidence-based practice (EBP), providing quantitative insights into muscle activity and functional status. This emphasizes the importance of sEMG in biomechanical and kinematic research, as well as in the assessment of the effectiveness of physical therapy and other rehabilitation interventions. Therefore, sEMG provides an excellent platform for interdisciplinary learning in physical therapy education.

The integration of sEMG technology within the STEM disciplines provides students with an enriched learning environment where theoretical knowledge is complemented by practical application. For example, the meticulous collection and rigorous analysis of sEMG data enable students to attain a profound comprehension of the relation between biomechanics and the underlying physiological mechanisms of muscle activity (9), exemplifying the application of Science principles. The application of technology is exemplified by the utilization of sEMG equipment and the proficient operation of data processing software. Engineering principles are applied through the interpretation and strategic application of sEMG signals, including the customization of rehabilitation programs for targeted muscle groups, such as adjusting exercise prescriptions based on sEMG signals to optimize patient outcomes (10). The recording of sEMG signals includes the following steps: (i) detection of myoelectric potentials with surface electrodes (bipolar electrode pairs, electrode arrays, or grids), (ii) amplification of these potentials, (iii) analog filtering of amplified potentials to prevent aliasing, and (iv) converting analog to digital signals by sampling (11). In the realm of engineering, we collaborated with biomedical engineers to develop a sEMG device specifically designed for physical therapy education. This device features a user-friendly interface and a lightweight design, enhancing the accuracy and efficiency of data collection (10). Mathematics also plays an integral role in the analysis of sEMG data, wherein students are required to apply statistical methods and computational algorithms to interpret the data, thereby reaching scientifically valid conclusions (12). For instance, we calculated the root mean square (RMS) values and mean/median frequencies of sEMG signals, which involves mathematical concepts in signal processing. Additionally, EMG features have been recently obtained from both linear and nonlinear analysis methods (13). These mathematical models aid in a more precise understanding of the relationship between muscle activity and motor performance for us to predict the effectiveness of rehabilitation training (14).

## 3 Application of sEMG technology in physical therapy education

sEMG technology has gained recognition as a core tool in physical therapy education, with its increasing adoption resulting in a transformation of traditional teaching models and curriculum structures. In this evolution, sEMG technology serves not merely as an element of the teaching content, but more significantly, it cultivates the development of students' critical thinking and practical skills.

Current physical therapy curriculum design, as exemplified by the standards set by the American Physical Therapy Association (APTA), emphasizes the inclusion of the fundamental theory of sEMG technology, signal acquisition, data analysis, and clinical applications (15). By engaging with the principles of sEMG, students enhance their comprehension of muscle electrophysiological activity and master essential skills in signal measurement and processing. This curriculum development process highlights the interdisciplinary integration of STEM fields—Science, Technology, Engineering, and Mathematics—thereby equipping students with a comprehensive, holistic framework of knowledge. Consequently, students are better prepared to apply sEMG technology in their future careers, facilitating the delivery of personalized and precise rehabilitation treatments.

Educators are also increasingly incorporating innovative teaching methods, such as laboratory practice, flipped classrooms, simulation software, and interactive teaching tools, with the objective of enhancing students' practical knowledge and abilities. These pedagogical approaches encourage student engagement and active participation (4). Through collaborative discussions and hands-on activities, students exhibit enhanced precision in analyzing muscle movements and interpreting sEMG signal features (2). This pedagogical shift not only serves to reinforce students' theoretical knowledge but also markedly enhances their abilities in clinical application.

For instance, the incorporation of sEMG into the curriculum has led to improved precision in muscle activity analysis, a skill that is directly applicable in the design of personalized rehabilitation plans. As demonstrated in a local physical therapy curriculum in Chile, students who engaged with sEMG technology showed a comprehensive understanding of the physiological basis of muscle activity and an enhanced capacity to interpret sEMG parameters through practical exercises (2). Furthermore, students have shown greater accuracy in using sEMG technology for monitoring the effects of rehabilitation training, a skill that is essential for clinical assessment (16). These enhancements in educational methodology have not only reinforced theoretical knowledge but also significantly improved students' abilities in the clinical application of sEMG technology (17).

Furthermore, physical therapy curriculums can also include a comparative study of sEMG technology with other bioelectric signal technologies, such as electrocardiography (ECG) and electroencephalography (EEG) (18). This comparative approach enables students to develop critical evaluation skills to assess the clinical value of technology while considering its potential limitations. By cultivating critical thinking, students are thus better prepared to discerningly select and apply the appropriate technologies such as sEMG in clinical practice with greater precision.

## 4 Barriers to the integration of sEMG technology in physical therapy education

Despite the considerable potential of sEMG technology in physical therapy education, its practical application is hindered by a number of challenges. Firstly, the expenses associated with technology and equipment can be prohibitive and constrains the capacity educational institutions to integrate sEMG technology, particularly in regions with limited resources. For example, Manzur-Valdivia and Alvarez-Ruf (16) observed that in developing countries such as Chile, the broad integration of sEMG is impeded by the cost, availability, and portability of the equipment. Moreover, the complex nature of the equipment requires a robust foundation in technical knowledge from the operators, which can be challenging for those with limited experience. Specifically, a lack of proficiency with regard to signal processing and data analysis may result in erroneous interpretations and misapplications of sEMG technology (11).

Secondly, educational systems frequently lack the requisite resources and expertise to integrate sEMG technology seamlessly into the curriculum. For example, research conducted by Felici and Del Vecchio (19) revealed that even leading global academic institutions have significant shortcomings in conveying the fundamental theories of muscle neural control within their exercise physiology courses. The survey findings indicated that a limited number of courses address the governing principles of EMG at the individual motor unit level, which has resulted in inadequate student comprehension and a diminished appreciation of the clinical application value of sEMG technology. Campanini et al. (17) have also observed that the lack of doctoral programs in physical therapy and occupational therapy in numerous countries greatly impedes the cultivation of specialized talent and obstructs academic research and professional advancement in sEMG technology. The lack of such advanced degree programs diminishes the academic sector's ability to pursue sophisticated research competencies, consequently restricting the extensive application of sEMG technology in both clinical practice and scientific inquiry.

To effectively address these challenges, it is recommended that a suite of targeted initiatives be implemented. In addressing challenges pertaining to technology and equipment, the development of economical alternatives should be pursued in conjunction with manufacturers. Alternatively, existing low-cost technologies, such as smartphone applications, could be leveraged to facilitate the collection and analysis of sEMG data. Furthermore, educational curricula must encompass comprehensive training on the operation of sEMG equipment to mitigate technical challenges and ensure students are able to utilize equipment proficiently (20).

Regarding educational and economic challenges, the ability of educational institutions to procure sEMG technology may be augmented through the provision of financial support from governmental and private entities. Moreover, online education platforms and open-source resources provide flexible and cost-effective solutions, facilitating broader student engagement with sEMG technology. For example, open educational websites provide free instructional materials and laboratory exercise guides on sEMG technology, allowing students to gain knowledge of sEMG without the need for expensive equipment (3).

Specific tools and tutorials are particularly beneficial for the post-processing analysis of EMG signals and for gaining a deeper understanding of physiological principles. In this context, we refer the readers to the comprehensive teaching materials provided by Roberto Merletti, which are accessible at https://www.robertomerletti.it/en/emg/material/teaching/. These materials, licensed under a Creative Commons Attribution-NonCommercial 4.0 International License, offer a valuable educational resource for both students and professionals in the field. Additionally, the Journal of Electromyography and Kinesiology publishes a range of tutorial materials that provide in-depth knowledge and practical guidance for those seeking to enhance their skills in electromyography (7, 9, 21).

It is therefore imperative that there be interdisciplinary collaboration to advance the application of sEMG technology within physical therapy education. Through collaboration with engineers, data scientists, and other medical professionals, more suitable sEMG tools and teaching methodologies for educational settings can be developed with greater precision and intention. For example, the development of user-friendly sEMG data analysis software in collaboration with computer scientists, can streamline the processing of data, thereby facilitating student comprehension and the application of sEMG data (22). However, it is essential to underscore that a solid grasp of knowledge of several basic concepts from anatomy, neurophysiology, and physics, among others is required. The user needs to properly locate the electrodes and be aware of particularities depending for example on the muscle evaluated (23). Similarly, sEMG users must understand the nuances of signal quality, including the ability to distinguish between good and bad signals, to ensure that the analysis conducted is both accurate and meaningful (24).

The implementation of these initiatives will enable the field to effectively overcome the challenges associated with the application of sEMG technology in physical therapy education and establish a robust foundation for its expanded utilization.

## 5 Innovation in education and curriculum development

To ensure the effective integration of sEMG technology into the existing educational system, it is essential that curricula undergo continual innovation and adaptation. The integration of sEMG technology must extend beyond mere theoretical instruction, with an emphasis on enhancing students' technical proficiencies through hands-on practice and practical application.

Firstly, educational curricula should construct teaching modules centered on sEMG technology, which must encompass the following core components: basic theory, equipment operation, data analysis, and clinical applications. In this manner, students will gain a comprehensive understanding of sEMG technology from multiple perspectives and, through practical experience, develop the fundamental competencies required to excel in this field.

Educational curricula must be designed to create teaching modules focused on sEMG technology, taking a balanced approach that covers the essential aspects without overstepping into territories typically reserved for engineering expertise.

Physical therapists need to integrate math and physiology knowledge which is key to interpreting sEMG data, as well as familiarized with proper knowledge related to data acquisition, signal processing, analysis and clinical applications knowledge to understand and use sEMG in their professions and guide rehabilitation programs and evaluate treatment outcomes.

While a basic comprehension of concepts like amplifier function and signal processing is beneficial, the physiotherapy curriculum aims to prepare students to effectively utilize sEMG technology in clinical practice, rather than to achieve a level of technical proficiency that would be more fitting for an engineer. This approach ensures that physiotherapists are well-equipped to apply sEMG in their work, maintaining a clear demarcation between their clinical skills and the more specialized engineering skills.

Secondly, interdisciplinary collaboration represents a promising avenue for advancing education innovation. It is recommended that physical therapy courses engage with other disciplines, such as engineering, computer science, and data analytics with the aim of co-creating comprehensive teaching projects (25). Such an interdisciplinary approach not only facilitates a more holistic educational experience but also enhances students' comprehension of the diverse applications of sEMG technology across various fields.

Furthermore, educational curricula should incorporate contemporary teaching methods, such as problem-based learning (PBL), case studies, and interactive instructional tools, which have demonstrated a proven track record in enhancing the effectiveness of sEMG technology teaching (16, 26–28). By employing these methodologies, students develop the capacity to learn and apply sEMG technology within the context of addressing real-world challenges, thereby enhancing their critical thinking and clinical reasoning abilities (29).

It is also imperative that policymakers and educational institutions collaborate to guarantee that curricula are aligned with clinical practice needs and that systematic teaching of sEMG technology is instituted through multi-level educational strategies. This necessitates the provision of foundational knowledge of sEMG technology at the undergraduate level, with the opportunity for further exploration of advanced concepts within graduate courses (30).

In conclusion, to assess and enhance the STEM capabilities of physical therapists, educational curricula must integrate quantitative and qualitative evaluations regarding the practical implementation of sEMG technology. Ongoing assessments coupled with constructive feedback can ensure the continuous improvement of educational content and methods that align with the evolving demands of clinical practice.

The implementation of these initiatives will facilitate the integration of sEMG technology, which will positively impact the overall quality of education by enhancing students' proficiency in technical skills, critical analysis, and clinical judgment. This will establish a robust platform for the cultivation of physical therapy professionals endowed with STEM literacy.

## 6 Case studies and empirical research

The application of sEMG technology in the field of physical therapy education has yielded substantial evidence through numerous case studies and empirical research. These studies have provided substantial evidence of the efficacy of sEMG technology within specific educational contexts and have contributed to a profound comprehension and assessment of teaching methodologies (2, 3, 16).

An exemplary case can be observed in the undergraduate physical therapy curriculum in Chile, which has markedly enhanced student engagement and comprehension of human movement through the integration of sEMG technology with active learning strategies (16). As a result of their various laboratory activities, students acquired a comprehensive understanding of the physiological basis of sEMG. Furthermore, these students were also able to enhance their capacity to interpret the intensity of sEMG signals through practical exercises. This holistic pedagogical approach has demonstrated consistent efficacy across diverse student cohorts, thereby affirming its extensive applicability within educational settings (2).

The free 3-day Winter School program hosted by sEMG researchers in Brazil serves as another illustrative case study, in which participants' understanding of sEMG technology was significantly enhanced through an integrated approach combining theoretical and practical course content. The critical evaluative skills of participants were evaluated through written examinations, with the outcomes demonstrating notable advancements in their comprehension and assessment of sEMG technology (2).

However, while early course-based engagement in sEMG training is prominent, a notable shortfall within Chilean physical therapy schools is the development of practical skills during clinical internships. This underscores the imperative for educational curriculum design to more closely integrate the theory and practice of sEMG technology, thereby augmenting students' capabilities in practical clinical applications (19).

The result of these case studies and empirical research emphasize the pivotal role of educational innovation in advancing the pedagogical application of sEMG technology. Moreover, they highlight the necessity of maintaining methodological rigor, which is an essential factor of enhancing the quality and credibility of the research.

## 7 Prospects and recommendations

The application of sEMG technology in physical therapy education is a crucial step in fostering clinical professionals with a comprehensive understanding of STEM principles.

To this end, it is recommended that educational policymakers revise the physical therapy curriculum standards to include the fundamental principles and proficient application techniques of sEMG technology as mandatory content. Furthermore, it is also recommended that educational institutions are encouraged to partner with technology companies to incorporate state-of-the-art equipment, providing students with opportunities for more hands-on experiences.

At the same time, educators should actively participate in sEMG technology training and incorporate it into their teaching designs. The use of case studies and clinical simulations can facilitate the enhancement of students' practical skills and clinical reasoning abilities. Furthermore, educators should encourage students involvement in research projects involving sEMG technology to foster the integration of academia and practice.

Similarly, students should concurrently leverage digital resources, such as online courses and virtual laboratories, to augment their theoretical knowledge and practical skills. They should also proactively search for and engage with research projects to cultivate innovative thinking and the capacity to target complex problems. The implementation of these strategies will provide a robust foundation for the efficient integration and application of sEMG technology in physical therapy education.

Through the adoption of these initiatives, the integration of sEMG technology into physical therapy education will consequently enrich the learning experience and prepare students to distinguish themselves in the rapidly evolving healthcare landscape, where technology and clinical practice are increasingly intertwined.

## 8 Educational innovation and curriculum development

As sEMG technology becomes an integral part of enhancing STEM literacy in physical therapy education, it is imperative to outline the foundational knowledge that undergraduates should possess. To fully grasp and apply sEMG concepts, students should have a solid understanding of basic exercise physiological principles, particularly those related to muscle and nerve function. A background in fundamental electrical circuits and signal processing would also be beneficial, as these concepts are integral to the operation and interpretation of sEMG devices. Additionally, a basic knowledge of statistics is essential for analyzing sEMG data effectively. By ensuring that the curriculum prerequisites students to have these foundational sciences and mathematical skills, incorporation sEMG technology into education framework enhances students' understanding of biomechanics, physiology, and data analysis, aligning with STEM principles.

Gizzi L and Felici F proposed a syllabus for teaching basic STEM concepts and their applications in sEMG, which includes the content of an interdisciplinary academic course on neuromechanics that requires an understanding of physics and electrophysiology for undergraduate students. It is believed that this syllabus may provide a viable pathway for implementing a stronger emphasis on STEM and evidence-based approaches in certain fields, such as physiotherapy or sports science (31).

While it is crucial to integrate STEM principles, including sEMG technology, into physical therapy education, the timing of such integration is a significant consideration. We have carefully examined the feasibility of introducing sEMG at the undergraduate level and believe it is not only feasible but also beneficial. An introductory understanding of sEMG, focusing on basic theory and practical application, can be effectively incorporated into the undergraduate curriculum. This early exposure provides students with a foundational knowledge that is essential for their future studies and professional practice.

For more advanced topics, such as sophisticated data analysis and the development of customized rehabilitation plans using sEMG data, we propose that postgraduate education is a more appropriate setting. This advanced training can build upon the foundational skills acquired at the undergraduate level, enabling students to further explore the advanced applications and intricacies of sEMG technology.

We have ensured that our recommendations are aligned with the educational goals of providing a progressive and comprehensive learning experience. By introducing sEMG technology at the undergraduate level and expanding on this knowledge in postgraduate studies, we can prepare physical therapists who are well-versed in the latest diagnostic and therapeutic tools.

## 9 Conclusion

This paper presents a comprehensive analysis of the integration of sEMG technology in physical therapy education, alongside the introduction of novel teaching strategies. Notwithstanding the limitations in resources and scope of this study, it makes a substantial contribution to educational practice. Further research is required to investigate the applicability and effectiveness of sEMG technology in various educational settings, with particular consideration of the cost-effectiveness and accessibility of the technology. We urge educators and policymakers give due consideration to the integration of sEMG technology into the STEM education framework and provide support for interdisciplinary collaboration with the goal of equipping the next generation of physical therapists with innovative capabilities and critical thinking skills. By leveraging these initiatives, we can ensure that physical therapy education progresses in harmony with clinical practice, thereby providing patients with higher quality treatment.

In summary, the potential for the application of sEMG technology in physical therapy education is vast, but it demands collaborative efforts of educational policymakers, teachers, students, and the entire industry to achieve its in-depth integration and widespread application within the educational system. It is anticipated that the implementation of these measures will catalyze a revolutionary transformation in the field of physical therapy education, providing future healthcare professionals with deeper and more efficient learning opportunities.
